# 

**Published:** 1892-05

**Authors:** 


// HALL'S
### JOURNAL OF HEALTH

TRUTH DEMANDS NO SACRIFICE; ERROR CAN MAKE NONE.

Vol. 39. MAY, 1892. No. 5.

### A WORD WITH OUR READERS.

Some six years ago we took upon ourself the management of HALL'S JOURNAL OF HEALTH, because we believed we could be of service to our fellow men through its instrumentality. We have done our best with this aim constantly before us. We then set our mark at 50,000 readers. These figures, although large, were not extravagant, in view of the very moderate subscription price of the Journal and the amount of information of value to the home and family, contained in every monthly issue, information which, if heeded, would save to the subscriber many times the subscription price, and much physical suffering.

Now, at this stage of our endeavors, is it asking too much of our constant yearly patrons to request of them, one and all, to so far interest themselves in our work, as to bring it to the attention of their friends and neighbors, and thus make the endeavor to procure for us one additional subscriber in each case. If this were successfully done, we should soon arrive at our gauge of merited patronage, leaving us no excuse for failing to furnish our readers with the BEST FAMILY HEALTH JOURNAL IN EXISTENCE, if it is not so now. Who will say us nay?

### LITERARY.

### THE WORLD'S COLUMBIAN EXPOSITION.

Send 50 cents to Bond & Co., 576 Rookery, Chicago, and you will receive, post paid, a four hundred page advance Guide to the Exposition, with elegant engravings of the Grounds and Buildings, Portraits of its leading spirits, and a Map of the City of Chicago ; all of the Rules governing the Exposition and Exhibitors, and all information which can be given out in advance of its opening. Also, other Engravings and printed information will be sent you as published. It will be a very valuable Book and every person should secure a copy.

BACTERIOLOGICAL DIAGNOSIS. By James Eisenberg, Ph. D., M. D., Vienna. Translated, etc., from the 2d German Edition, by Norval H. Pierce, M. D., Chicago. 4to 184 pp. The F. A. Davis Co., Philadelphia, Publishers. Price, $1.50, net.

The German edition of this comprehensive summary of a long and purely scientific study of those infinitesimal promulgators of disease, is dedicated to Doctor Robert Koch, the renowned discoverer of the Lymph cure for consumptives, which created such a profound sensation some months ago, the author of the work under review having, whilst his pupil, made most of his experiments at the Koch laboratory. The work consists mainly of the classification, habits, growth and characteristics of the 138 distinct micro-organisms critically examined and studied, and to health officers and physicians will be found of great value. In Europe it has won its way as a standard authority.

LETTERS TO FARMERS' SONS. By Henry S. Chase, M. D. St. Louis, published by the Twentieth Century Co.

This compact little volume is devoted largely to political economy, and such practical questions of the day as enter into the life of every citizen, fairly presented and discussed.

The veteran author and reformer extensively known as "Pa" Chase, has a book in press by the "Purdy Pub. Co.," of Chicago, called "The Dignity of Sex," which explains the origin and evolution of sex, the ethics of marriage, and the law of appetite, the laws of heritage and control of sex. The author had something valuable to say, and has said it in simple, strong, honest, clean language. "Pa" Chase stands for the freedom of woman, equally with the freedom of man.

We are in receipt of the

FIRST DECENNIAL CATALOGUE OF THE COLLEGE OF PHYSICIANS AND SURGEONS OF CHICAGO.

The new requirements for admission, in addition to proofs of good moral character, necessitate a diploma from a literary or scientific school granting the degree of B. S. or B. A., or their equivalent, as shown under examination. These rules, if rigidly enforced, will tend to the elevation of the profession in ways greatly needed.

DELSARTIAN PHYSICAL CULTURE; illustrated by Carrica Le Favre. Fowler & Wells Co., Publishers. Paper, 25 cents, extra cloth, 75 cents.

This is one of the completest treatises of the kind that has come under our notice; if followed out in practice great benefit would be derived from it.

AMATIVENESS. ADVICE TO MARRIED AND SINGLE. Fowler & Wells Co., 775 Broadway.

The contents of this volume are explained by its title. The information and advice given will be found of great value to those who are thoughtful enough to make it the rule of their lives.

THE May *Century,* in addition to the serials now running, will contain complete stories by Thomas Nelson Page and Wolcott Balestier, and a humorous skit by Harry Stillwell Edwards, author of "Two Runaways." Also Señor Castelar's Life of Columbus will be commenced and run through several numbers

Our thanks are tendered for the following treatises :

Second Year's Work in Class diseases at the N. Y. Post Graduate Hospital. By Charles D. Kelsey, M. D.

Tobacco, Insanity and Nervousness. By Dr. L. Bremer, St. Louis.

Aphasia Due to Sub-dural Hemorrhage, etc., by the same author.

Treatment of Laryngeal Phthisis. By Robert Levy, M. D., Denver, Col.

---

### MRS. LILLIE M. SPENCER, ARTIST.

There are few patrons of art who are unfmiliar with the works of the celebrated artist whose name heads this paragraph. The writer recalls the circumstance when, years agone, a fresh young girl, she was discovered, so to speak, in a far away western cottage, whose interior walls were ornamented by crude charcoal sketches, the scintillations of her budding genius, which under the fostering care of appreciative friends, grew into the full blown artist, whose masterly achievements have found their way, well nigh the world round, and drawn to her the admiration of all lovers of high art.

A few years since Mrs. Spencer was led to seek a home in the Highlands bordering upon the Hudson, in the vicinity of Pouhgkeepsie, where her studio and handicraft at once took rank among the enticements of that picturesque region. This is what she says of this highland retreat, which she has christened.

### ROCK NEST.

Near the broad Hudson's
Shining breast,
High on the cliffs is built
Our Rock Nest.
Near to the skies as its name implies,
Above the city's cares, vanities and snares

If fruit and flowers among
Of varied hue
Your weary self would hold
Communion true
With nature, in her grandest mood,
In love expressed,
Come ! and be welcome
At our pretty Rock Nest.

O dear Rock Nest,
Be ever blest,
And always prove
A place of rest
For aching heart
And weary brain,
And make the sad face
Smile again.

